# Self-Assembly and Regrowth of Metal Halide Perovskite Nanocrystals for Optoelectronic Applications

**DOI:** 10.1021/acs.accounts.1c00651

**Published:** 2022-01-17

**Authors:** Jiakai Liu, Xiaopeng Zheng, Omar F. Mohammed, Osman M. Bakr

**Affiliations:** † Division of Physical Sciences and Engineering, KAUST Catalysis Center (KCC), King Abdullah University of Science and Technology (KAUST), Thuwal 23955-6900, Kingdom of Saudi Arabia; ‡ College of New Materials and New Energies, Shenzhen Technology University, Shenzhen 518118, China

## Abstract

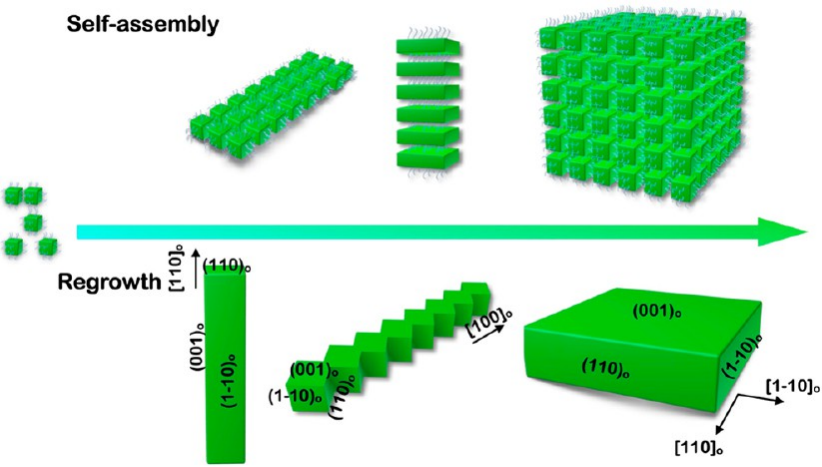

Over the past decade, the impressive development of metal halide perovskites (MHPs) has made them leading candidates for applications in photovoltaics (PVs), X-ray scintillators, and light-emitting diodes (LEDs). Constructing MHP nanocrystals (NCs) with promising optoelectronic properties using a low-cost approach is critical to realizing their commercial potential. Self-assembly and regrowth techniques provide a simple and powerful “bottom-up” platform for controlling the structure, shape, and dimensionality of MHP NCs. The soft ionic nature of MHP NCs, in conjunction with their low formation energy, rapid anion exchange, and ease of ion migration, enables the rearrangement of their overall appearance via self-assembly or regrowth. Because of their low formation energy and highly dynamic surface ligands, MHP NCs have a higher propensity to regrow than conventional hard-lattice NCs. Moreover, their self-assembly and regrowth can be achieved simultaneously. The self-assembly of NCs into close-packed, long-range-ordered mesostructures provides a platform for modulating their electronic properties (e.g., conductivity and carrier mobility). Moreover, assembled MHP NCs exhibit collective properties (e.g., superfluorescence, renormalized emission, longer phase coherence times, and long exciton diffusion lengths) that can translate into dramatic improvements in device performance. Further regrowth into fused MHP nanostructures with the removal of ligand barriers between NCs could facilitate charge carrier transport, eliminate surface point defects, and enhance stability against moisture, light, and electron-beam irradiation. However, the synthesis strategies, diversity and complexity of structures, and optoelectronic applications that emanate from the self-assembly and regrowth of MHPs have not yet received much attention. Consequently, a comprehensive understanding of the design principles of self-assembled and fused MHP nanostructures will fuel further advances in their optoelectronic applications.

In this Account, we review the latest developments in the self-assembly and regrowth of MHP NCs. We begin with a survey of the mechanisms, driving forces, and techniques for controlling MHP NC self-assembly. We then explore the phase transition of fused MHP nanostructures at the atomic level, delving into the mechanisms of facet-directed connections and the kinetics of their shape-modulation behavior, which have been elucidated with the aid of high-resolution transmission electron microscopy (HRTEM) and first-principles density functional theory calculations of surface energies. We further outline the applications of assembled and fused nanostructures. Finally, we conclude with a perspective on current challenges and future directions in the field of MHP NCs.

## Key References

- LiuJ.; SongK.; ShinY.; LiuX.; ChenJ.; YaoK. X.; PanJ.; YangC.; YinJ.; XuL.-J.; YangH.; El-ZohryA. M.; XinB.; MitraS.; HedhiliM. N.; RoqanI. S.; MohammedO. F.; HanY.; BakrO. M.Light-Induced Self-Assembly of Cubic CsPbBr_3_ Perovskite Nanocrystals into Nanowires. Chem. Mater.2019, 31, 6642–6649.^1^*In this work, the light-induced synthesis of CsPbBr_3_ nanowires through the regrowth of nanocrystals (NCs) was studied, with a systematic investigation of their phase transition, shape evolution, anisotropic growth mechanism, and growth preference.*
- PanJ.; LiX.; GongX.; YinJ.; ZhouD.; SinatraL.; HuangR.; LiuJ.; ChenJ.; DursunI.; El-ZohryA. M.; SaidaminovM. I.; SunH.-T.; MohammedO. F.; YeC.; SargentE. H.; BakrO. M.Halogen Vacancies Enable Ligand-Assisted Self-Assembly of Perovskite Quantum Dots into Nanowires. Angew. Chem., Int. Ed.2019, 131, 16223–1622710.1002/anie.20190910931529587.^2^*This work explored a halide-vacancy-driven regrowth mechanism of metal halide perovskite (MHP) NCs and provided insights into the corresponding defect-correlated dynamics and defect-assisted fabrication of devices.*
- ZhangY.; SunR.; OuX.; FuK.; ChenQ.; DingY.; XuL. J.; LiuL.; HanY.; MalkoA. V.; LiuX.; YangH.; BakrO. M.; LiuH.; MohammedO. F.Metal Halide Perovskite Nanosheet for X-ray High-Resolution Scintillation Imaging Screens. ACS Nano2019, 13, 2520–25253072102310.1021/acsnano.8b09484.^3^*This work reported the self-assembly of MHP nanosheets for application in X-ray high-resolution scintillation detectors.*
- LiuJ.; SongK.; ZhengX.; YinJ.; YaoK. X.; ChenC.; YangH.; HedhiliM. N.; ZhangW.; HanP.; MohammedO. F.; HanY.; BakrO. M.Cyanamide Passivation Enables Robust Elemental Imaging of Metal Halide Perovskites at Atomic Resolution. J. Phys. Chem. Lett.2021, 12, 10402–104093467258810.1021/acs.jpclett.1c02830.^4^*This work reported the ligand-induced regrowth of CsPbBr_3_ NCs into nanoplates via an interface-assisted technique. The obtained nanoplates exhibited ultrahigh stability against electron irradiation and were elementally mapped via atomic-resolution X-ray energy dispersive spectroscopy.*

## 1 Introduction

Constructing nanomaterials with a desired structure and function is an aim of nanotechnology. The spontaneous arrangement of individual components into organized structures, that is, self-assembly and regrowth, is one of the most facile approaches for achieving this goal.^5−7^ The self-assembly of nanocrystals (NCs) into larger, long-range-ordered macroscopic arrays,^8,9^ superlattices,^10,11^ and larger crystals^12^ can result in a set of unique properties, including enhanced mechanical strength,^13^ electronic couplings,^14^ improved charge-carrier transport,^15−17^ and superior stability,^18^ compared with those of the individual constituents of such organized structures. Therefore, these macroscopic assembled nanostructures have stimulated the development of a wide range of applications in optoelectronic and thermoelectric devices and catalysis.^15,19^ Moreover, the bottom-up self-assembly and regrowth strategy provides a simple but effective platform for producing diverse NC ensembles, in contrast to top-down techniques that require elaborate facilities and produce limited structures.^5,9^

**Scheme 1:**
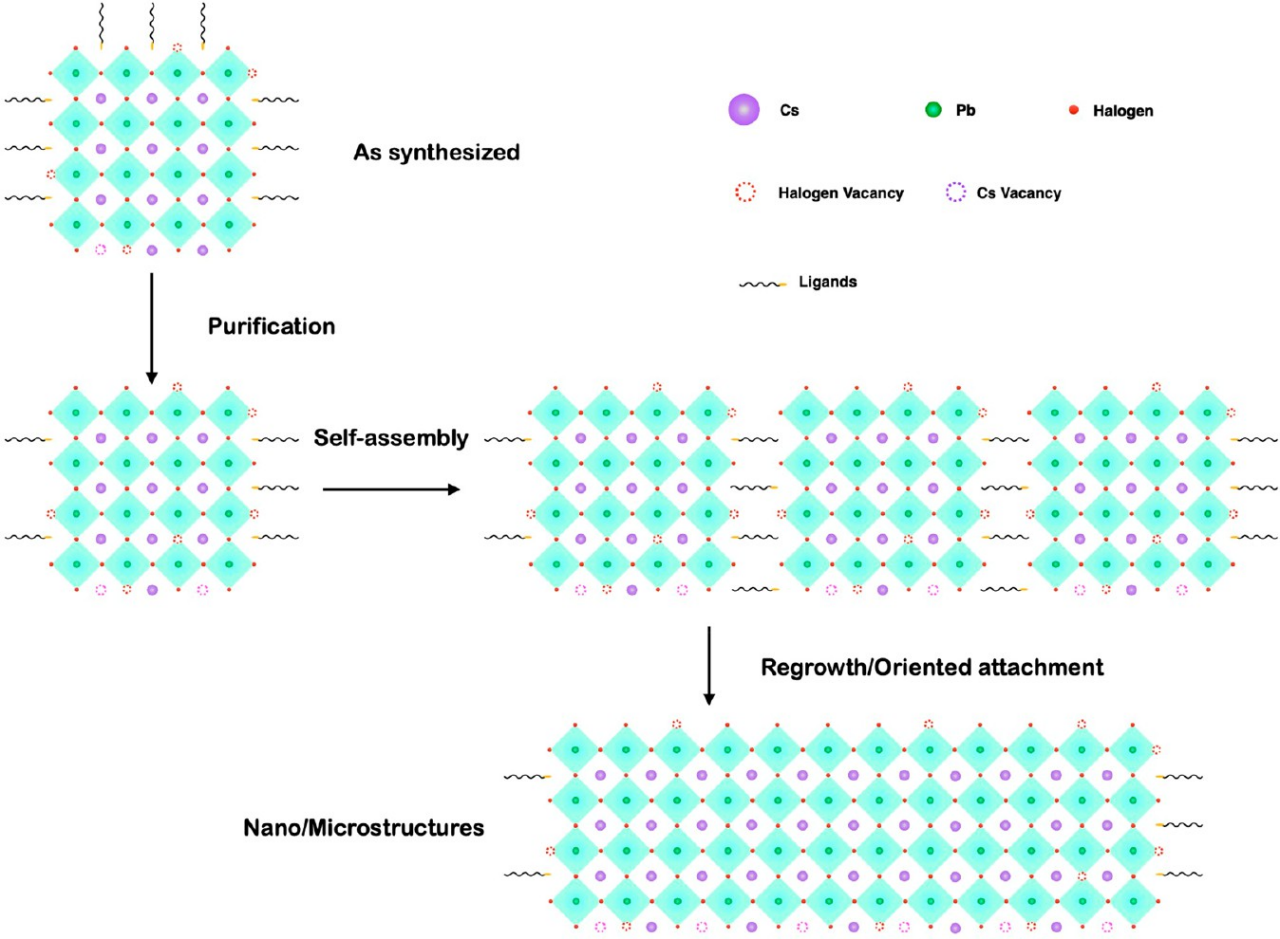
Schematic of the Self-Organization of Metal Halide Perovskite (MHP) Colloidal Nanocrystals (NCs) into Highly Ordered Superlattices and Their Regrowth into Large Microstructures

Unlike traditional chalcogenide NCs, metal halide perovskite (MHP) NCs are soft ionic materials that possess unique features,^20−23^ such as highly dynamic surface ligands,^24,25^ rapid anion exchange,^26−28^ and ease of ion migration,^29^ which facilitate their regrowth and the rearrangement of their overall appearance (Scheme 1). Consequently, the regrowth of MHP NCs can occur after the self-assembly process.^1,30^ The organization and fusion of MHP NCs, which are ideally suited for self-assembly and regrowth, into targeted nanostructures has been pursued as one method to modulate their optoelectronic properties. One example is self-organized three-dimensional (3D) superlattices, which exhibit key signatures of superfluorescence with red-shifted photoluminescence (PL)^31,32^ and a long exciton diffusion length.^3,33^ These closely packed superlattices with long-range order accommodate a high density of exciton states of low energetic disorder and a long dephasing time, enabling the construction of macroscopic quantum states.^34^ Importantly, the electronic properties of NCs, such as their conductivity and carrier mobility, can be substantially modulated when they are assembled into close-packed structures with strong NC coupling, which facilitates charge transport and is beneficial for fabricating high-performance devices. Moreover, the fused MHP nanostructures can lower the defect density and thus exhibit considerably enhanced stability against moisture,^35,36^ light,^37^ and electron-beam irradiation^4,38^ compared with their individual NC counterparts, offering a route for improving the inherent vulnerability that plagues MHPs. Consequently, these nanostructures have been employed in fabricating light-emitting diodes (LEDs),^32^ X-ray scintillators,^3^ and lasers^34^ and have potential applications in nanoantennas^39^ and photoelectric-compatible quantum processors.^34^ However, MHP self-assembly and regrowth have not been as intensively studied as MHP NC syntheses. Thus, the mechanisms of facet-directed connections and shape modulation have not yet been clearly elucidated. Therefore, many of the finer details regarding the self-assembly and fusion mechanisms of NCs and their unique roles in optoelectronic devices remain unclear. Summarizing the current progress in MHP NC self-assembly and regrowth and their property implications would be beneficial in stimulating further research efforts toward realizing the full potential of these highly ordered materials in optoelectronic applications.

In this Account, we review the following topics concerning advances in the self-assembly and regrowth of MHP NCs: the mechanisms, driving forces, and techniques for controlling the assembly process; morphological and phase evolution at the atomic level; investigations of oriented attachment mechanisms; the potential applications of assembled and fused nanostructures. We conclude by discussing the current challenges facing this field and forecasting possible opportunities.

## 2 Self-Assembly of MHP NCs

**Figure 1:**
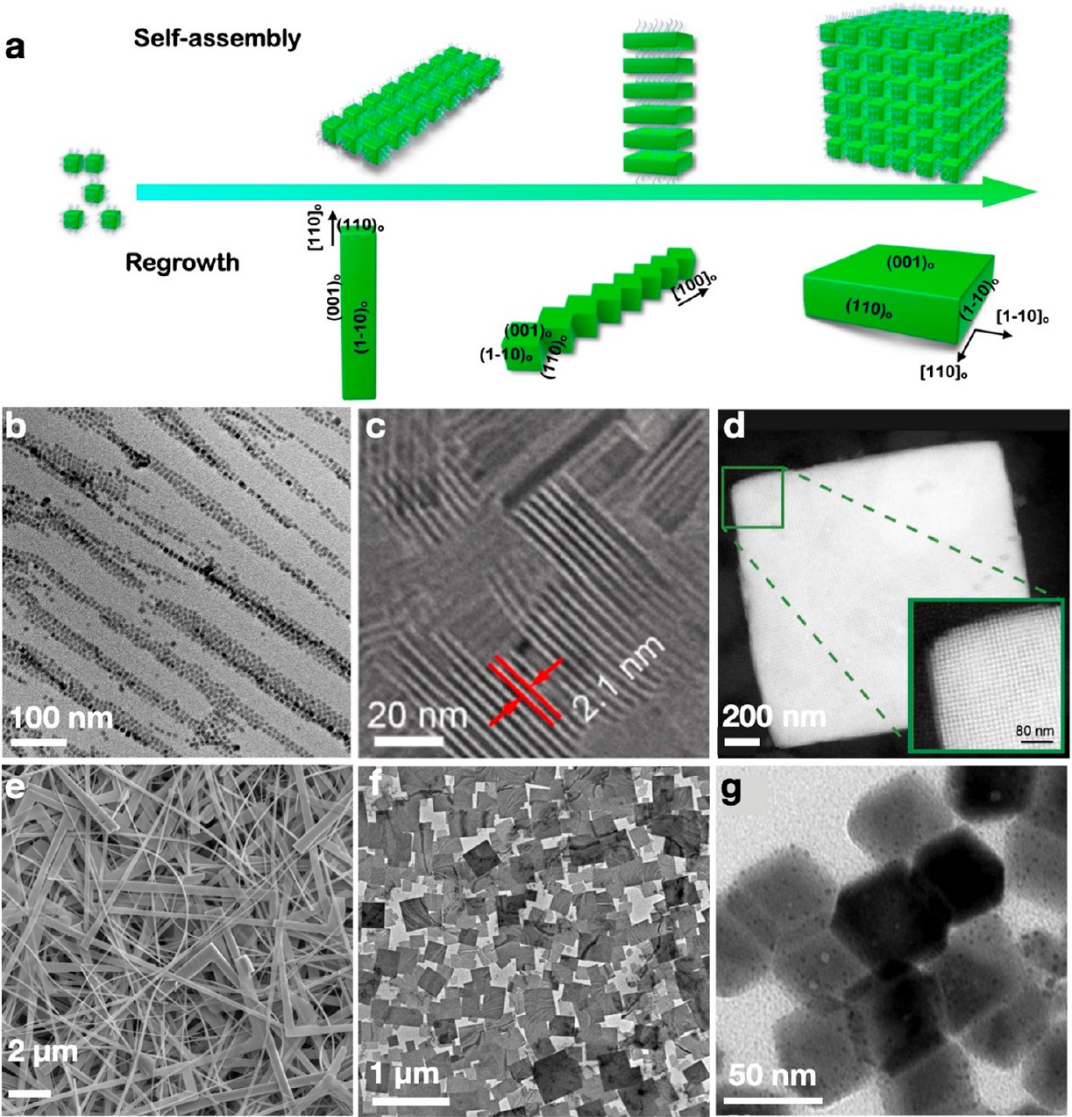
(a) Schematic of the self-organization of MHP colloidal NCs into highly ordered superlattices (b–d) and further regrowth into large, bulky crystals (e–g). (b) One-dimensional (1D) superlattice chains. Reproduced with permission from ref (1). Copyright 2019 American Chemical Society. (c) Two-dimensional (2D) layered superlattices. Reproduced with permission from ref (3). Copyright 2019 American Chemical Society. (d) Three-dimensional (3D) superlattice. Reproduced with permission from ref (31). Copyright 2018 Nature Publishing Group. (e) Nanowires. Reproduced with permission from ref (1). Copyright 2019 American Chemical Society. (f) Nanoplates. Reproduced with permission from ref (4). Copyright 2021 American Chemical Society. (g) Nanocuboids. Reproduced with permission from ref (40). Copyright 2019, Wiley.

**Figure 2:**
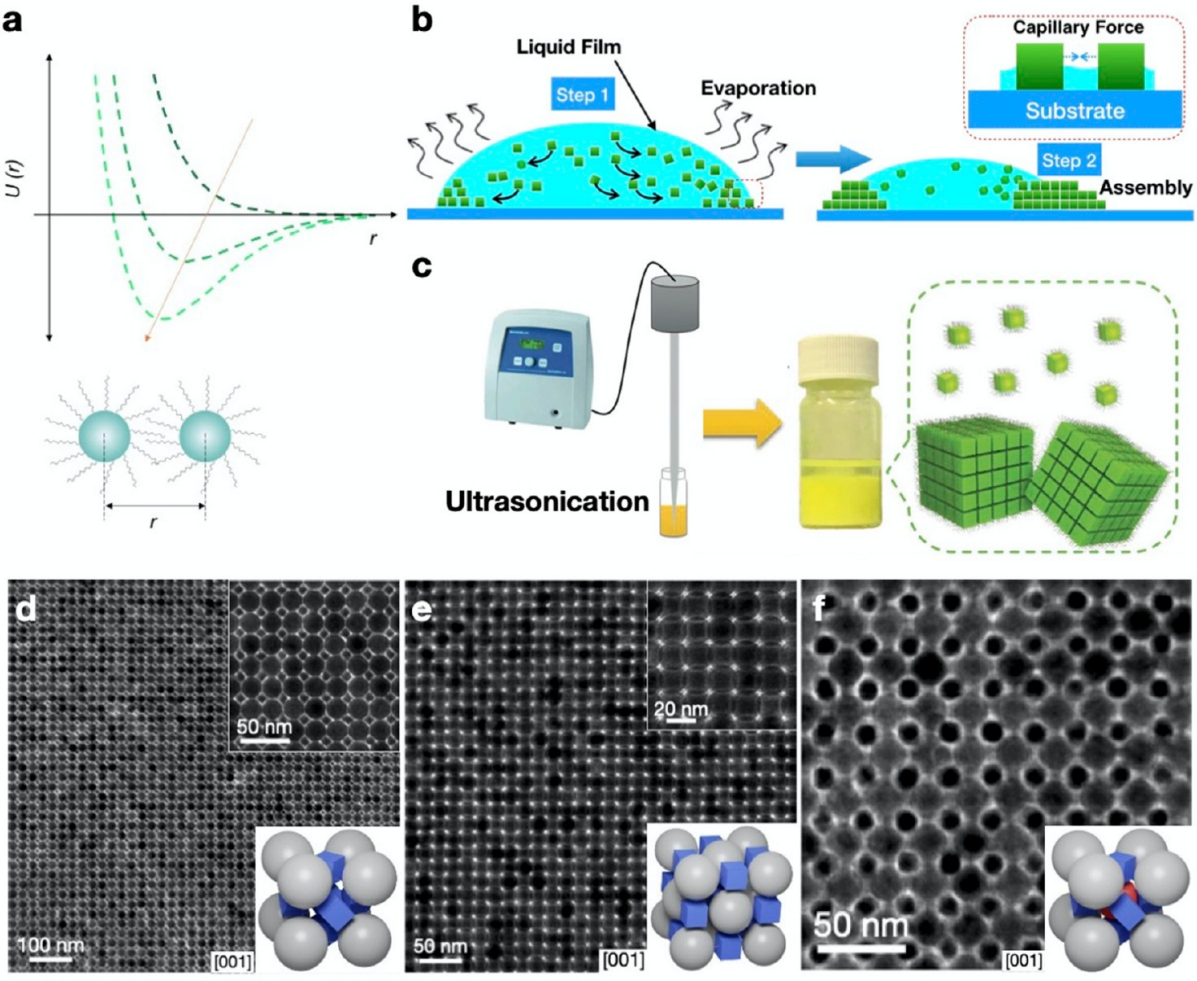
(a) Evolution of the effective pair interaction potential, *U*, at different self-assembly stages. Reproduced with permission from ref (5). Copyright 2016 American Chemical Society. (b) Scheme depicting the capillary forces associated with solvent evaporation. (c) Schematic illustration of the formation of self-assembled 3D CsPbBr_3_ superlattices by ultrasonication. Reproduced with permission from ref (32). Copyright 2018 Wiley. (d–f) TEM images of binary ABO_3_-type (d), binary NaCl-type (e), and ternary ABO_3_-type (f) superlattices from MHP nanocubes. Reproduced with permission from ref (47). Copyright 2021 Nature Publishing Group.

Self-assembly is a spontaneous process that organizes individual components into orderly nanostructures, e.g., one-dimensional (1D) superlattice chains,^1^ two-dimensional (2D) layered superlattices,^3^ and 3D superlattices,^27^ as shown in Figure 1b–d. The self-assembly is driven by NC**–**NC interactions, including van der Waals forces between inorganic cores and between surface ligands, as well as osmotic, electrostatic, and elastic contributions.^5^ The balance of these forces can be illustrated by the effective interparticle pair interaction potential, *U* (Figure 2a). In colloidal nanoparticle solutions, the repulsive potential dominates and favors the monodispersion of the nanoparticles (Figure 2a, dark-green trace). During the self-assembly process, the effective interparticle interaction changes from repulsive to attractive (Figure 2a, light-green trace). The total removal of solvent results in the curdling of the NCs into a superlattice, with a balance between ligand elastic repulsion and van der Waals attractive forces.

van der Waals forces are speculated to be the most dominant interaction at the nanoscale, usually acting in a manner that brings particles together. As one of the three types of van der Waals forces, dipole–dipole interactions dominate the initial stage of self-assembly because of the long interaction distance of dipolar attractions.^41^ Inorganic MHPs (i.e., CsPbX_3_), in which a dipole moment should not intrinsically exist, have exhibited a perfect distortion-free cubic structure.^1^ Electric polarization can emerge from the disruption of crystal symmetry, which is a process that involves the migration of A cations, the movement of B cations away from the center of the BX_6_ octahedra, and distortion of the BX_6_ octahedra, all of which can induce a dipole moment.^42^ When a polar solvent (i.e., ethanol) induces CsPbI_3_ lattice distortion, the adsorption of polar molecules causes the migration of Cs^+^ as well as distortion of the PbI_6_ octahedra, resulting in the breaking of symmetry and the polarization of the CsPbI_3_ NC.^43^ When a third polarized CsPbI_3_ NC approaches two polarized CsPbI_3_ NCs, the arrangement along the rectilinear direction would create the smallest gradient of the dipole potential field, which accounts for linear alignment into a 1D superlattice chain. This characteristic is conspicuous in organic–inorganic hybrid perovskites (i.e., MAPbX_3_), in which the asymmetry of organic cations results in the absence of an inversion center in the structure.^42,44^

Self-assembly of NCs is commonly triggered by solvent evaporation or by varying the polarity of the reaction system and destabilizing the NC capping ligands. Figure 2b shows the preparation of assembled nanostructures by evaporation of the colloidal solvent. During solvent evaporation, the interparticle distance decreases, and NCs can potentially assemble in an orderly manner to maximize the total entropy of the system. A strong capillary interaction is further exerted to drive parallel alignment between neighboring NCs (Figure 2b).^19^ Once the NCs are closely spaced, they start to align and stack at the interface to form superlattices. The formation of a superlattice via drying-mediated self-assembly can be modulated by (i) the initial NC concentration, (ii) the temperature of solvent evaporation, and (iii) the concentration of the capping ligands.^45^ For example, the addition of oleic acid and oleylamine (OAm) can passivate bare NC surfaces, prevent solvent dewetting, and stimulate depletion attraction, thereby assisting in the formation of CsPbBr_3_ NC superlattices,^31^ where the building blocks (CsPbBr_3_ nanocubes) are atomically aligned exclusively via the four vertical {100}_c_ facets^46^ (subscript “c” represents cubic crystal structure). The solvent evaporation technique has also been extended to the construction of binary and ternary superlattices via coassembly of cubic CsPbBr_3_ NCs and other types of NCs (e.g., Fe_3_O_4_, NaGdF_4_, and PbS),^47^ enriching the membership of MHP ensemble families (Figure 2d–f).

The assembled nanostructures are strongly affected by the interactions between the solvent and the capping ligands.^48,49^ By exploiting the dynamic ligand–surface interaction of MHPs and their sensitivity to solvent polarity, researchers have used appropriate solvents to trigger self-assembly. In these assemblies, solvent selection is critical, and a uniform stable dispersion of NCs is undesirable. For example, CsPbBr_3_ NCs can disperse stably in toluene but tend to arrange into chain-like assemblies in hexane.^50^ This arrangement occurs because when MHP NCs are dispersed in a nonpolar solvent such as hexane, the excess aliphatic ligand complex with ionic species forms via oleophilic interactions, and alkyl ligand chains connect to one another through strong van der Waals interactions, resulting in the self-assembly of CsPbBr_3_ NCs into 1D superlattice chains.

In addition to the aforementioned investigations of the contributions of solvent evaporation and polarity, efforts have been dedicated to investigating the anisotropic and isotropic assembly of MHP NCs, including surfactant interactions,^51,52^ external forces^32,53^ (e.g., sonication in Figure 2c), and template-assisted assembly.^54^ For example, prepatterned polydimethylsiloxane templates were used for the template-induced self-assembly of CsPbBr_3_ NCs into large-area 2D supercrystals.^54^

Because of the inherently soft ionic nature of the MHP crystal structure and low formation energy, the obtained MHP superlattices tend toward continuous regrowth into large crystals (Figure 1e–g). Although the ligands render MHP NCs resistant to aggregation, these ligands loosely attach to the surface of MHP NCs because of the highly dynamic binding between the surface-capping ligands and the oppositely charged NC surface ions. Therefore, in contrast to most conventional NCs (e.g., chalcogenide), whose self-assembly preferentially ends with superlattices,^45^ MHP NCs self-assemble and regrow simultaneously into different nanostructures (e.g., Figure 1b,e)^1,30^ but are particularly prone to regrowth.

## 3 Phase Transition, Morphological Evolution, and Mechanism of MHP NC Regrowth

Regrowth is commonly driven by thermodynamics and occurs when NCs are physically attached. Surface atoms usually exhibit higher chemical reactivity than interior atoms because of the considerable number of dangling bonds between surface atoms. Removing surface ligands exposes NC surfaces, and this process is associated with a substantial increase in the attraction potential among NCs; consequently, the NC units in a superlattice can continue to grow (Scheme 1).

**Figure 3:**
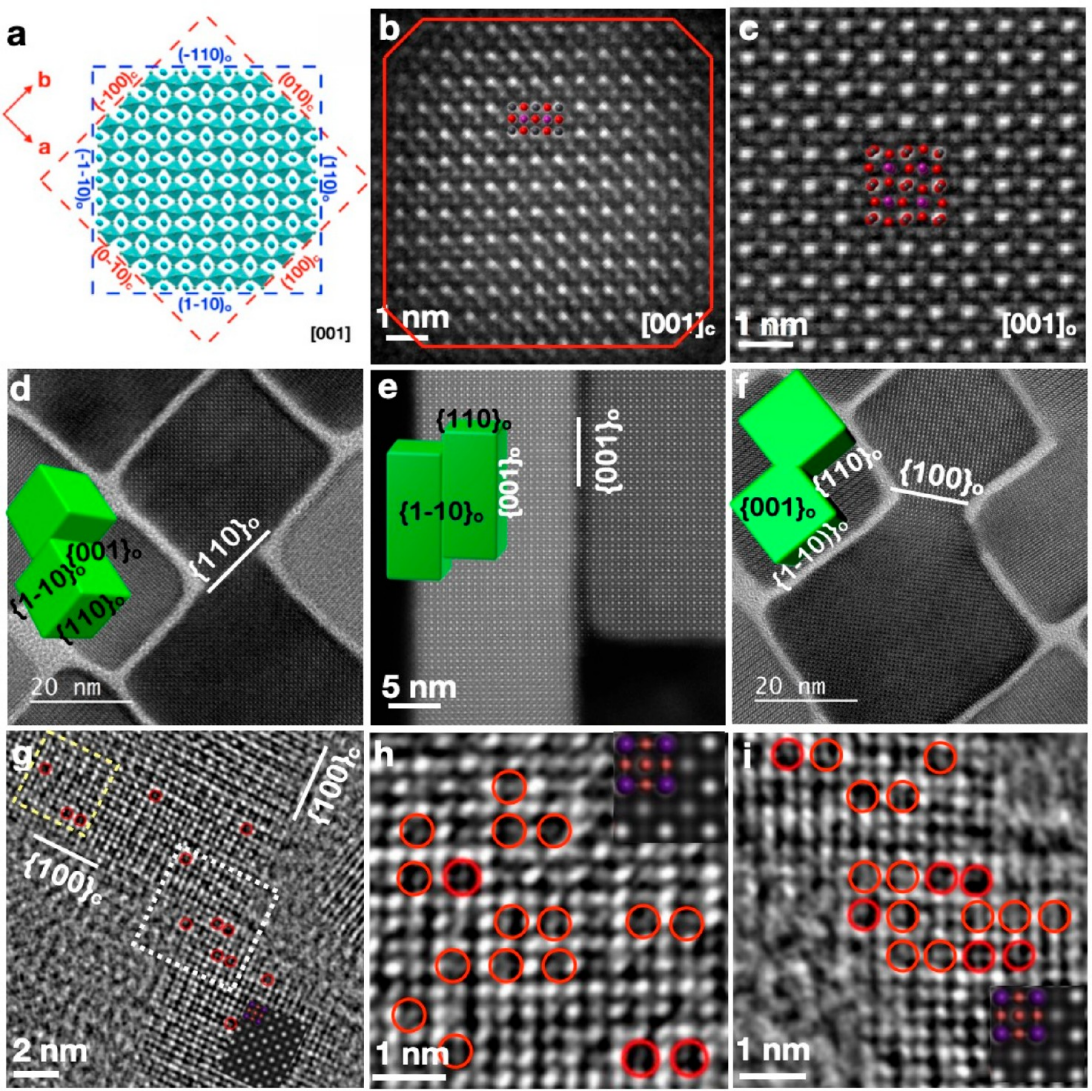
(a) Geometrical relationship between cubic and orthorhombic unit-cell axes and faces. (b) Atomically resolved high-angle annular dark-field scanning transmission electron microscopy (HAADF-STEM) image of cubic-phase CsPbBr_3_ NCs viewed along the [001]_c_ zone axis and (c) regrowth of orthorhombic-phase CsPbBr_3_ bulk crystals viewed along the [11̅0]_o_ zone axis. (d–f) High-resolution transmission electron microscopy (HRTEM) images showing the coalescence of CsPbBr_3_ NCs via oriented attachment along the [110]_o_ (d), [001]_o_ (e), and [100]_o_ (f) crystallographic directions. (g–i) Investigation of the vacancy distribution in initial CsPbBr_3_ NCs. Inset is a simulated HRTEM image of CsPbBr_3_. Red circles represent Br vacancies. Panels b–e reproduced with permission from ref (1). Copyright 2019 American Chemical Society. Panels f–i reproduced with permission from ref (4). Copyright 2021 American Chemical Society.

The regrowth of MHP derivatives is usually accompanied by a phase transformation. However, the atomic structure of MHP NCs remains poorly understood. For example, whether the crystal structure of CsPbBr_3_, which is the most prevalent type of MHP NC, is cubic or orthorhombic remains a topic of debate.^55,56^ The orthorhombic phase evolves from a slight tilting of the PbBr_6_ octahedra in the cubic structure, which preserves the 3D network of corner-sharing octahedra while introducing structural differences between axially and equatorially coordinated halides. This small tilting of the PbBr_6_ octahedra cannot be distinguished by powder X-ray diffraction analysis. Aberration-corrected scanning transmission electron microscopy (STEM) has thus been used to elucidate the atomic details of the crystal structure of CsPbBr_3_ NCs. The high-angle annular dark-field (HAADF)-STEM image shown in Figure 3b indicates that CsPbBr_3_ NCs are of a cubic phase (ICSD 29073; *Pm*3̅*m* (221); *a* = 0.5874 nm) and exhibit truncated cubic shapes. During the regrowth process, the initial cubic CsPbBr_3_ NCs undergo a phase transformation from the cubic phase to the thermodynamically more stable orthorhombic phase (ICSD 97851, *Pbnm* (62), *a* = 0.8207 nm, *b* = 0.8255 nm, *c* = 1.1759 nm), as displayed in Figure 3c. A similar phenomenon has been observed among iodized derivatives.^43^

With respect to the isotropic cubic phase, understanding the alignment of the orthorhombic structural axes is paramount for elucidating their anisotropic shape evolution (e.g., into nanowires and nanoplates). Orthorhombic CsPbBr_3_ NCs are modeled as possessing four side {110}_o_ facets (subscript “o” represents orthorhombic crystal structure), two bottom {001}_o_ facets, and 12 edge facets (four {100}_o_ and eight {112}_o_ facets).^57,58^ Density functional theory (DFT) calculations indicate that both the {001}_o_ and {110}_o_ surfaces of CsPbBr_3_ NCs are terminated with a CsBr surface.^59^ However, the surface Cs^+^ ions are inclined to be replaced with surfactants;^60^ this replacement process, combined with abundant surface Br^–^ vacancies,^61^ leads to the possible exposure of PbBr_2_ termination. Undoubtedly, the coexistence of CsBr- and PbBr_2_-terminated surfaces ensures continuous regrowth, which has been confirmed by atomic-resolution STEM.^1^ The relatively higher surface energy of the {100}_o_ surfaces terminated with CsPbBr^2+^ and Br_2_^2–^ facets makes these surfaces less stable than the {001}_o_ and {110}_o_ surfaces (surface energies {100}_o_ > {110}_o_ > {001}_o_).^4^ Different surface-atom configurations of the NC surfaces are particularly important because they can determine the preferred growth direction.

Oriented attachment (OA), one of the most important mechanisms for controlling NC growth, is becoming a prevalent approach for controlling the design of nanostructures. NCs in a dispersed colloidal solution collide frequently because of Brownian motion; however, not all of these collisions result in NC attachment. Only NCs that share a common crystallographic orientation can undergo an effective collision.^62^ Otherwise, the NCs undergo continuous rotations until they match with a perfect lattice.^63^ Afterward, the NCs undergo oriented attachment at the contacted facets with a common crystallographic orientation.^62^ As illustrated in Figure 3d–f, MHP NCs can coalesce in various ways, including face-to-face (e.g., [110]_o_ or [001]_o_ direction), edge-to-edge (e.g., [100]_o_ direction), and even corner-to-corner.^1,4,64^ Even if the {100}_o_ surfaces are thermodynamically less supported, the electrostatic interaction that originates from their charged character (facets terminated with CsPbBr^2+^ and Br_2_^2–^) can promote the likelihood of edge-to-edge coalescence. Long-distance interactions (e.g., van der Waals forces and Coulombic interactions) are critical to bringing nanoparticles sufficiently close together for OA.^65^ For instance, in a colloidal solution, if two MHP NCs are far away from each other, the primary driving force for OA is van der Waals interactions, while interatomic Coulombic interactions are negligible in comparison because the interatomic Coulombic interactions are screened. By contrast, when two MHP NCs are in close proximity, electrostatic interactions become dominant, driving the NCs to approach each other and eventually fuse.^65,66^ From a thermodynamic viewpoint, the fusion in a coherent crystallographic orientation eliminates the interfaces of the NCs; thus, a reduction in surface energy is assumed to be the thermodynamic driving force.^67^ The total energy change of MHPs, as soft ionic materials, is largely derived from the interatomic Coulombic interactions arising from both surface and interior atoms, where the former also contribute to a reduction in surface energy.^66^ Simulation^68^ and transmission electron microscopy (TEM) results revealed that the presence of abundant Br vacancies in MHP NCs (Figure 3g–i) may further promote the intrinsic Coulombic interactions.^4^ Because of dangling Pb bonds and a substantial concentration of vacancies on the surface of MHP NCs, their surface atoms exhibit high chemical reactivity toward the absorption of ions and contribute to their regrowth into various microstructures.^2^

After NCs attach, the interface region starts to regrow until the interface boundary completely disappears. In addition to the perfect perovskite structure, planar defects (e.g., Ruddlesden–Popper (RP) planar faults, symmetric grain boundaries (GBs), or asymmetric GBs)^69^ can also be introduced at the surface where attachment occurs. Defects form mainly because of the relatively fast dynamics of attachment, in conjunction with the low formation energy of MHPs, where coalescence can occasionally occur even if the NCs have not reached identical crystallographic orientations. For example, the merging of two NCs with the same terminated surface leads to a RP planar fault. If two NCs with the same surface termination do not coalesce in parallel, a symmetric GB will form. If the NCs are fused with different surface terminations, an asymmetric GB will form.

Shape evolution is known to be a kinetic process in which high-energy surfaces grow faster than low-energy surfaces.^70,71^ According to DFT calculations, the {001}_o_ facets maintain the densest atomic stacking mode and possess lower surface energy than the {110}_o_ facets. Consequently, the orientation with {001}_o_ facets and growth along the four [110]_o_ directions is the thermodynamically most favorable shape-evolution mode and ensures maximally exposed, low-energy {001}_o_ facets.^4^ Ideally, CsPbBr_3_ NCs evolve into nanoplates along the [110]_o_ directions.^4^ However, the effects of capping ligands, external forces, or assembly techniques, for instance, could alter the growth trajectory and lead to the development of other morphologies. For example, the contribution of the surfactant OAm accounts for the anisotropy associated with the production of nanowires.^1^ The basal (largest) facets of the nanowires are two {001}_o_ and two {110}_o_ facets, which are side nanowire facets, whereas the two remaining bottom planes are {110}_o_ planes. From a thermodynamic perspective, nanowires grow along the [110]_o_ direction to maximally expose low-energy {001}_o_ surfaces (Figure 3d). In addition, research on vacancy-assisted regrowth has revealed that the {100}_o_ surfaces possess a much larger Br-vacancy (V_Br_) formation energy than the {110}_o_ surfaces and that the higher vacancy density on the {110}_o_ facets leads to the growth of nanowires along the [110]_o_ direction (Figure 3d).^2^ Pradhan et al. reported that the direction of facet connection can be further tailored by controlling the reactant composition ratios.^72^ The inclination of Pb-rich (Br-deficient) NCs enables the connection of {100}_o_ edge facets (Figure 3f), resulting in zigzag 1D nanowires. However, Pb-deficient (Br-rich) compositions promote merging along the {110}_o_ facets, which indicates that Br^–^ ions assist in the elimination of the {100}_o_ active edge facets and allow for connection mostly via {110}_o_ facets.^72^ These observations could seem in contrast to the aforementioned Br-vacancy-induced growth along the [110]_o_ direction.^2^ Therefore, additional experimental and theoretical investigations (related to, for example, the selective adherence of capping ligands to facets) are needed to reconcile the various evident observations of MHP nanostructure formation. In general, ligands contribute to the growth kinetics, preferential orientation, and shapes associated with NC self-assembly. For PbS NCs, oriented attachment occurs exclusively via the (100) facets (those with lower surface energy) because oleate ligands are strongly bound to the (110) facets, preventing them from oriented attachment through that surface orientation.^45^ Unlike the strongly bound oleate ligands in PbS, the surface ligands in soft ionic perovskites are loosely attached and highly dynamic. This contrasting behavior offers a plausible explanation for the differing roles of ligands in the oriented attachment of MHP NCs (versus chalcogenide NCs), in which the higher-energy (100)_o_ facets are still involved in oriented attachment.

## 4 Applications of Assembled and Regrown MHP Nanostructures

The self-assembly and regrowth of MHPs has afforded their use in numerous promising applications, including the fabrication of high-performance LEDs,^32,73^ lasers,^34,49^ and X-ray scintillators,^3^ and they are appealing candidates for use in nanoantennas^39^ and photoelectric-compatible quantum processors.^34^ The self-assembly of NCs into close-packed assemblies results in more efficient electronic behaviors (e.g., improved conductivity and carrier mobility). In addition, the assembled MHP NCs exhibit novel optoelectronic properties (e.g., superfluorescence,^31^ renormalized emission,^32^ longer phase coherence times, and longer exciton diffusion lengths^3,33^) because of the electronic and physical coupling of NCs. Moreover, further regrowth into fused MHP nanostructures with the removal of ligand barriers between NCs could facilitate charge carrier transportation and enhance stability against moisture, light, and electron-beam irradiation by eliminating surface point defects, making them useful in practical applications. These attractive properties and concerns are addressed in this section, with a discussion of representative cases.

**Figure 4:**
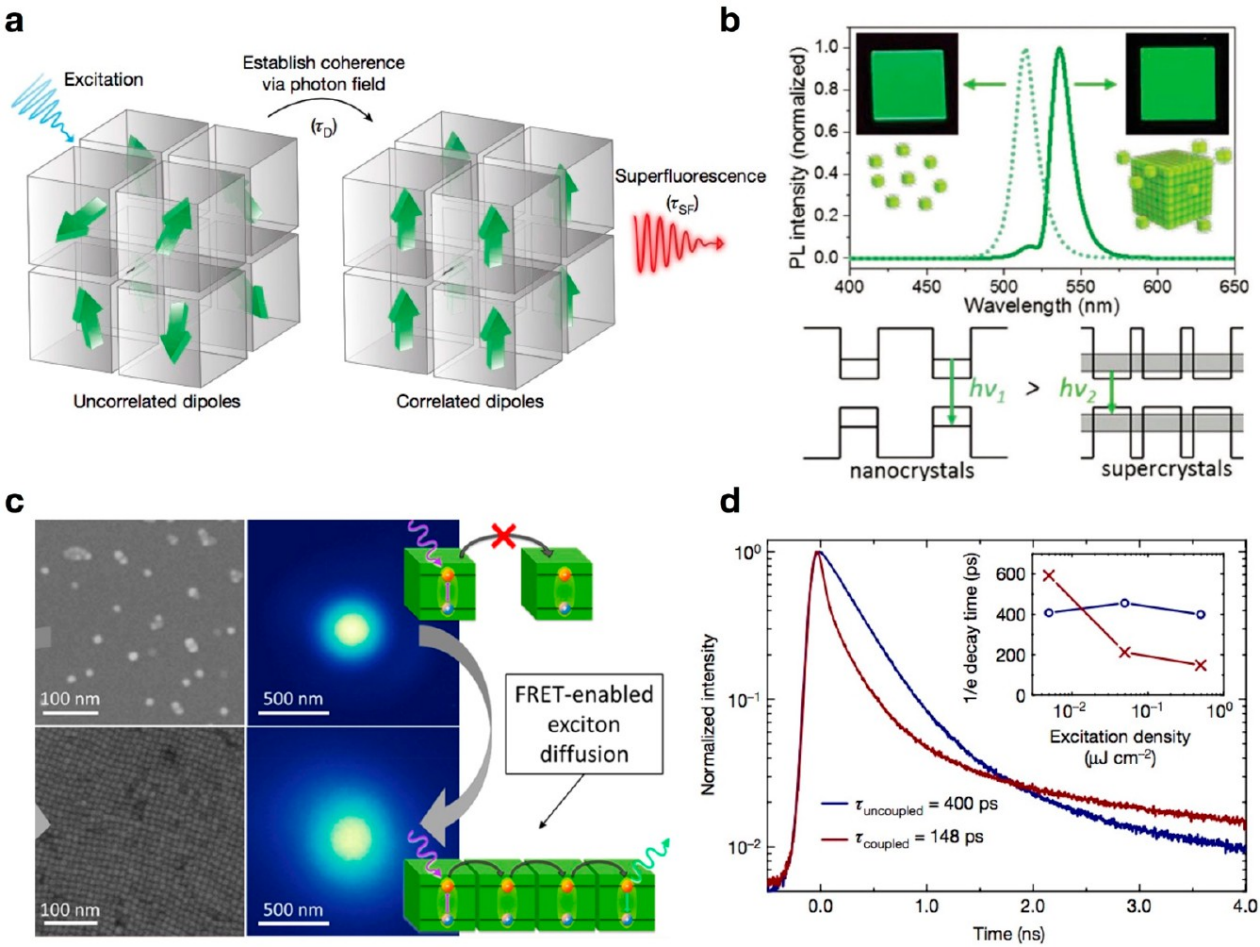
(a) Schematic of the build-up of superfluorescence in CsPbBr_3_ NC superlattice. Reproduced with permission from ref (31). Copyright 2018 Nature Publishing Group. (b) Optical properties and energy diagram of CsPbBr_3_ NCs and superlattices. Reproduced with permission from ref (32). Copyright 2018 Wiley. (c) Steady-state exciton diffusion measurement. Normalized profile of PL intensity emitted by sparse (top) and close-packed (bottom) MHP NC monolayer when excited with a diffraction-limited laser spot. Reproduced with permission from ref (33). Copyright 2019 American Chemical Society. (d) Time-resolved PL decay of uncoupled (blue) and coupled (dark red) NCs. Reproduced with permission from ref (31). Copyright 2018 Nature Publishing Group.

Assembled superstructures with novel properties arising from electronic coupling between NCs are expected to find applications in light-emitting devices. Compared with the individual NC building units, self-organized superlattices display obvious signatures of superfluorescence (short, intense bursts of light)^31^ (Figure 4a) because of the many-body quantum phenomenon induced by collective coupling; they also exhibit red-shifted narrowing emission^31,32,74^ (Figure 4b) with accelerated radiative decay (Figure 4d),^31,34,75^ longer phase coherence times, and fluorescence resonance energy transfer (FRET)-mediated long exciton diffusion lengths (Figure 4c).^3,33^

**Figure 5:**
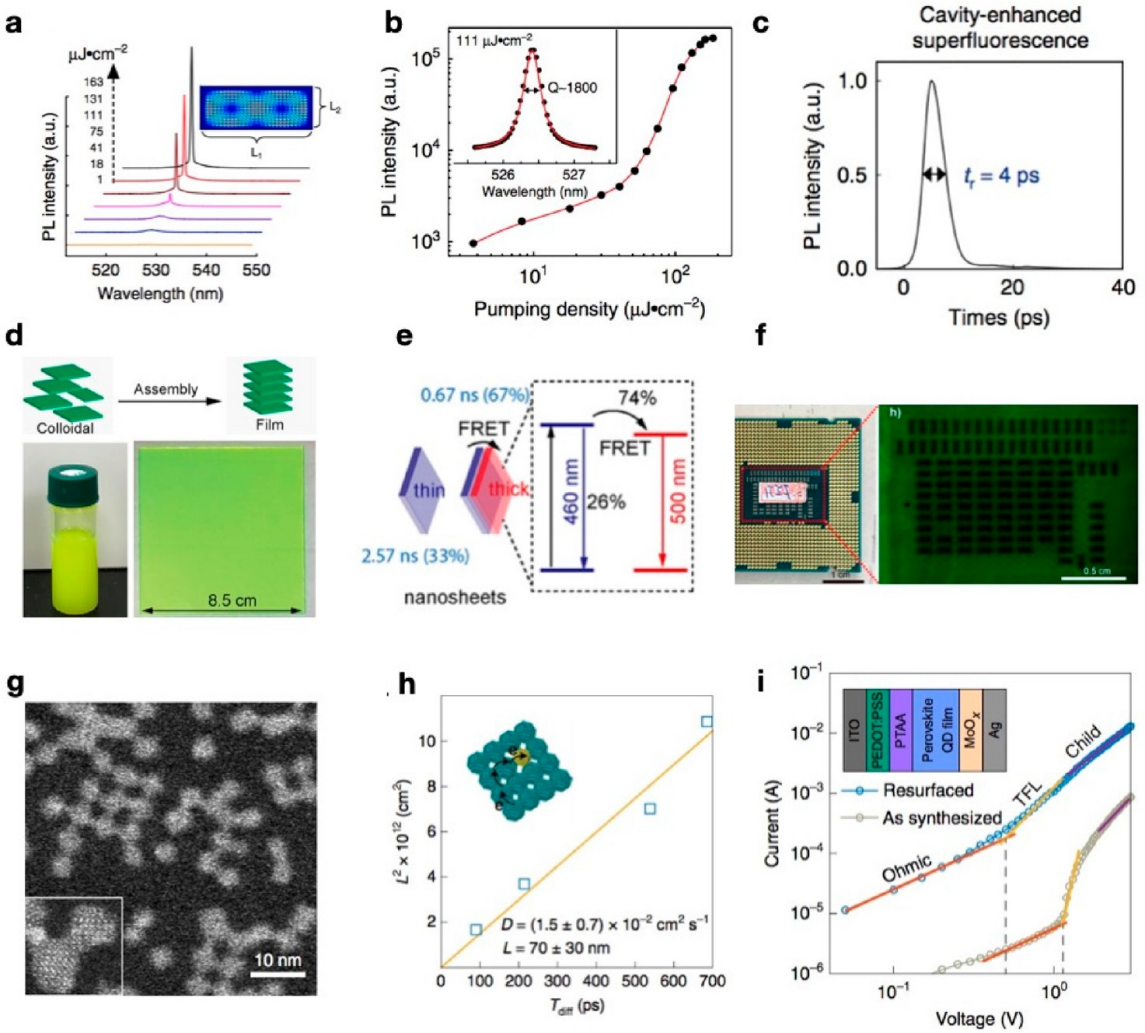
(a) PL spectra showing cavity-enhanced superfluorescence from a quantum-dot superlattice microcavity (QDSM). (b) Power dependence of the PL intensity in cavity mode. (c) Radiation dynamics. Panels a–c reproduced with permission from ref (34). Copyright 2020 Nature Publishing Group. (d) Schematic showing the self-assembly of CsPbBr_3_ nanosheets. (e) Schematic showing the energy transfer process from thin to thick nanosheets, with a fluorescence resonance energy transfer (FRET) efficiency of 74%. (f) Photographic and X-ray images of a standard central processing unit panel with a Si chip integrated underneath. Panels d–f reproduced with permission from ref (3). Copyright 2019, American Chemical Society. (g) STEM image revealing the self-assembly of CsPbBr_3_ NCs with an atomic-scale interparticle distance. (h) Measurement of the exciton diffusion length. (i) Current–voltage traces and carrier mobility measurements. Panels g–i reproduced with permission from ref (76). Copyright 2020 Nature Publishing Group.

To further exploit such superfluorescence characteristics (Figure 4a), Zhou et al. developed a perovskite-based quantum-dot superlattice microcavity (QDSM) that exhibits cavity-enhanced superfluorescence behavior and an optically stimulated amplification effect, as displayed in Figure 5a.^34^ The typical *Q*-factor of a QDSM can reach ∼2000 (Figure 5b). During the QDSM lasing process, the cavity field in the QDSM further accelerates the superfluorescence process, leading to a picosecond-scale radiative time (Figure 5c). Moreover, the coherent nature of these exciton states, which arises from the strong mesoscopic coupling between NCs, can enable the development of entangled multiphoton quantum light sources and may allow for the application of QDSMs in ultrafast, photoelectric-compatible quantum processors. However, decoupled multiquantum-well 2D superlattices, which exhibit a high photoluminescence quantum yield (PLQY), narrowband emission, and enhanced light outcoupling, have also emerged as desirable candidates for many practical applications, such as LEDs and nanoantennas.^39^

The self-assembly of MHP NCs into electronically coupled superlattices, resulting in the formation of a delocalized, extended electronic state, can renormalize their emission energy.^74^ These ensembles retain the high PL efficiency of their NC subunits, which can be exploited to develop a new set of electronic applications. For example, the assembled CsPbBr_3_ 3D superlattices exhibit red-shifted emission at 535 nm (Figure 4b), whereas the individual NCs emit cyan-green light (515 nm), allowing them to overcome the “green gap” and thus enabling the fabrication of efficient pure-green LEDs that satisfy the Rec. 2020 standard.^32^

Because of FRET (Figure 4c), CsPbBr_3_ NC assemblies exhibit extremely efficient exciton diffusion, demonstrating a record diffusion length of 200 nm with a diffusivity of 0.5 cm^2^ s^–1^; this performance is substantially better than that of chalcogen-based NCs.^3,33^ Their self-assembly in close-packed systems promotes communication between neighboring NCs by enabling the FRET of excitons, which results in the transport of excitonic energy in multiple steps before the excitons recombine. The occurrence of FRET in assemblies is critical for enhancing optoelectronic device performance. For example, an X-ray high-resolution scintillator fabricated from self-assembled CsPbBr_3_ nanosheets (Figure 5d) demonstrated markedly enhanced scintillation performance because of the energy transfer process inside the stacked nanosheets (Figure 5e), with a FRET efficiency of 74%. Such a simple prototype enabled spatial resolution within 0.2 mm (Figure 5f).^3^

Self-assembly is an effective method of surface engineering to achieve high-performance photovoltaics (PVs) and LEDs. The self-assembly of NCs into densely packed assemblies affords another level of modification for tailoring electronic properties (e.g., conductivity and carrier mobility). For example, a solvent-assisted assembly strategy can be used to modulate the prototypical, long, insulated ligands via surface engineering, leading to a close-packed and smooth surface.^77^ Connecting the NCs after removal of the surfactant barriers could considerably improve charge injection and carrier transport.^76,77^ Consequently, a substantially boosted external quantum efficiency (EQE) has been achieved, indicating considerable enhancement in the properties associated with the CsPbBr_3_ emitting layer, such as carrier transport and radiative decay.^77^ Furthermore, assisted by surface-functionalized self-assembly, an ultrasmooth monolayer nanocube thin film with a root-mean-square roughness of ∼4 Å has been fabricated.^78^ The short-chain ligands introduced into the system enable more efficient charge transport and substantially reduce the NC–NC interactions, thereby greatly promoting self-assembly. Similarly, a bipolar-shell-resurfacing strategy has been proposed to stabilize CsPbBr_3_ NCs, where ligand exchange leads to close-packed films with a long diffusion length, high carrier mobility, and reduced trap density.^76^ The TEM image in Figure 5g shows the features of assembled CsPbBr_3_ NCs. The contribution from FRET in the assembled films and the reduced trap density originating from a resurfaced bipolar shell led to elongated exciton diffusion lengths of ∼70 ± 30 nm (Figure 5h) and improved carrier mobility (Figure 5i). Self-assembled MHP NCs with atomic-scale interparticle distances and a resurfaced bipolar shell yielded EQEs of 12.3% and 22% for blue and green devices, respectively.^76^

**Figure 6:**
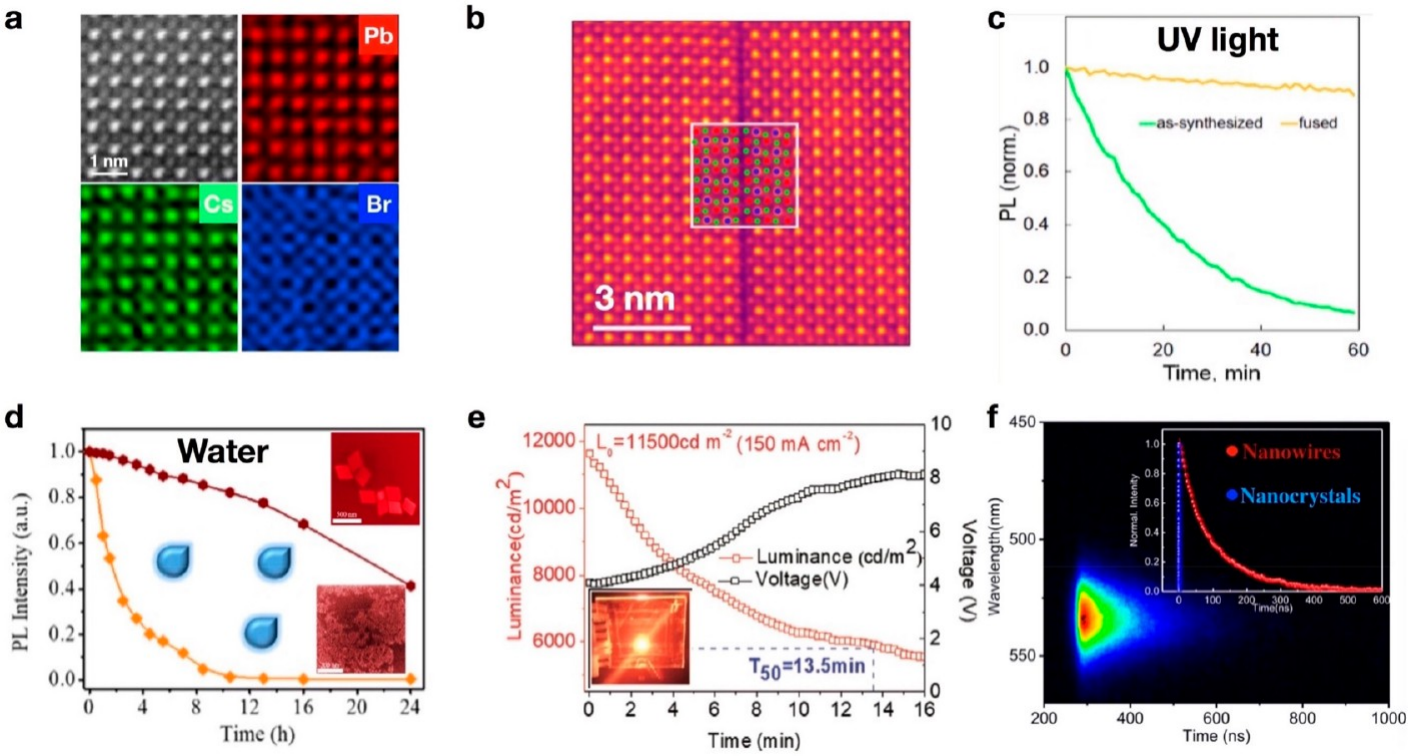
(a) HAADF-STEM image and atomic-resolution X-ray energy dispersive spectroscopy elemental mapping images of a CsPbBr_3_ nanoplate. Reproduced with permission from ref (4). Copyright 2021 American Chemical Society. (b) Atomic-resolution HAADF image of a Ruddlesden–Popper planar defect with an overlaid atomic model. (c) Normalized PL intensity dynamics of as-synthesized and fused CsPbBr_3_ NCs upon continuous ultraviolet (UV) light exposure. Panels b and c Reproduced with permission from ref (37). Copyright 2018 American Chemical Society. (d) Relative photoluminescence intensity of NCs and fused nanosheets immersed in water. Reproduced with permission from ref (35). Copyright 2020 American Chemical Society. (e) Performance of assembled nanowires for LEDs: a constant driving current of 6 mA (150 mA cm^–2^) led to an increase in luminance (*L*_0_) from 0 to 11500 cd m^–2^. The estimated operational half-lifetime (*T*_50_) at 100 cd m^–2^ was 694 h. Reproduced with permission from ref (73). Copyright 2020 Wiley. (f) PL lifetime measurements of CsPbBr_3_ NCs and the nanowires obtained through light-induced regrowth. Reproduced with permission from ref (1). Copyright 2019 American Chemical Society.

The inherent susceptibility of MHP derivatives to degradation remains a major obstacle to their practical application. Self-assembly and regrowth may offer a new avenue for overcoming their instability. For example, CsPbBr_3_ nanoplates obtained from the regrowth of their NCs exhibited ultrahigh stability under a 300 kV electron beam, and the first atomic-resolution X-ray energy dispersive spectroscopy elemental mapping data of MHPs were successfully acquired, as shown in Figure 6a.^4,38^ Similarly, fused CsPbBr_3_ nanoplates with RP defects (Figure 6b) showed substantially improved stability against UV light compared with as-synthesized NCs (see Figure 6c).^37^ Another example is the pressure-driven regrowth of MHP NCs into nanosheets (Figure 6d); compared with their NC units, these nanoplates demonstrate enhanced properties, including a higher PL intensity and remarkable resistance to water.^35^ The attenuation of surface defects and the disappearance of grain boundaries in perovskites by self-assembly and regrowth contribute to enhanced stability against moisture. In addition, a strategy was developed to fabricate 2D/3D perovskite films by the self-assembly of low-*n*-value 2D perovskite crystals with enhanced device stability against moisture.^36^ In summary, these results demonstrate that the stability of assembled or fused MHP derivatives is superior to that of unassembled NCs.

The formation of defect-free MHPs is key to the successful implementation of MHP nanostructures in optoelectronic devices.^21^ Surface point defects can be effectively self-healed during the self-assembly and regrowth process, resulting in low density of trap states in MHP nanostructures. For instance, self-assembled nanowire arrays are known to exhibit very high PLQY of 91% at a wavelength of 600 nm because of their low trap density and strong quantum confinement.^73^ This ultralow trap density could contribute to remarkable structural and environmental stability. As shown in Figure 6e, the fabricated LEDs based on these assembled nanowires exhibited a record luminance of 13644 cd m^–2^ with an EQE of 6.2%, along with substantially improved operational lifetimes (*T* = 13.5 min at 11500 cd m^–2^, *T* = 694 h at 100 cd m^–2^). In a similar study, fused highly crystalline nanowires demonstrated a carrier lifetime 2 orders of magnitude longer than that of the initial NCs (Figure 6f), which provides clear evidence that regrowth can reduce the defect concentration and thus hinder the nonradiative recombination in these fused CsPbBr_3_ nanowires.^1^

## 5 Conclusion and Outlook

In this Account, we focused on advances in the self-assembly and regrowth of MHP NCs, along with their assembly and fusion mechanisms, driving forces, preparation strategies, and potential applications. Undoubtedly, self-assembly and regrowth is a facile and powerful approach for controlling the structure, shape, and dimensions of MHPs. The novel optoelectronic properties (e.g., superfluorescence) of assembled MHP NCs, their promoted electronic behaviors, and their enhanced stability against electron-beam irradiation and light facilitate the fabrication of high-performance devices. Therefore, exploring the self-assembly and regrowth behavior of MHP NCs is important, and numerous opportunities lie ahead.

Self-assembled MHP NCs exhibit stronger coupling than conventional NC films, which enhances optoelectronic device performance. The electronic coupling of MHP NCs enables the high PLQY of the individual NCs to be retained, which can facilitate charge-carrier injection and reduce the probability of trap-assisted recombination.

The successful study of MHP NC regrowth provides a path toward comprehensively understanding the nucleation and growth kinetics of perovskite materials. The soft ionic nature of MHPs enables them to exhibit fast nucleation and growth kinetics such that the intermediate states during the dynamic growth process are undetectable by normal reaction-tracking approaches; consequently, the mechanism by which MHP NCs are formed remains unclear. The self-assembly and regrowth of MHP NCs can dramatically slow NC growth kinetics, enabling the tracking of NC trajectories. In addition, recently developed ultralow-dose electron microscopy techniques^79^ enable atomic-resolution imaging of MHPs to provide routes toward addressing the aforementioned uncertainties and fully elucidate the orientation growth process.

The formation of electron irradiation-stable MHP NCs via fusion provides a platform for studying defect species in perovskite semiconductors. Sufficient stability achieved against electron beams provides additional opportunities for much deeper investigations using TEM, such as research into the types of defects and mechanism of defect formation at the atomic scale. Understanding the origin of defects in MHP NCs is paramount in attaining long-term structural stability and improved optical efficiency.

Obstacles to fully understanding the complexities of self-assembly persist. First, the driving forces that control the formation of MHP assemblies remain largely unexplored; a fundamental analysis of these forces would assist the development of diverse assembled nanostructures. Accordingly, more attention should be devoted to investigating the strongly coupled MHP assemblies, which may provide avenues for new developments in optoelectronic devices with properties that exploit efficient charge transport and enhanced conductivity in those assemblies. However, predicting appealing assembled structures and phases will likely involve well-understood simulations (e.g., Monte Carlo and molecular dynamics simulations) coupled with experimental verification.
